# SYD1/444: Assessing the Readership of the UK National Database of Telemedicine

**DOI:** 10.2196/jmir.1.suppl1.e126

**Published:** 1999-09-19

**Authors:** J Briggs

**Affiliations:** ^1^University of Portsmouth, SouthseaUK

**Keywords:** Internet, Hypermedia, Readership Analysis, Telemedicine

## Abstract

**Introduction:**

The National Database of Telemedicine (NDTM) is a Web site containing information resources on UK telemedicine projects and related work. Its purpose is to provide a source of information to anyone researching the field or proposing a trial or a larger scale implementation of Telemedicine. The NDTM Web site was launched on 27th October 1998 and publicised extensively. Before and after its launch, the Web site has been kept up to date with information about new projects and modifications to the details kept about existing ones. It is clearly important for such a resource to keep track of who is reading it. We may need to tailor information to a particular audience, or bring the Web site to the attention of people who may not yet have found it. This paper describes a mechanism for tracking usage of the Web site that provides more information than conventional approaches.

**Methods:**

For technical reasons associated with the way the Internet works, the information that can be gathered about visitors to a page is severely limited. Counters can only tell you how many people have accessed a particular page - they do not tell you anything about those people. It is possible to find the Internet address of the visitor's computer and to use the domain name service (DNS) to translate this into a host name (e.g. www.dis.port.ac.uk). This provides some information about the visitor's origins. However obtaining information such as the visitor's name or email address is not possible. There are other factors that affect the ability to collect information, including how the user has configured their browser. However, by seeking the co-operation of the browser, it is possible to glean more information. The paper describes how we did this.

**Results:**

We have tracked usage of the Web site. The peak of visits was immediately after the launch but since then we have averaged about 120 visits per week. Where identifiable, the majority of these are from within the UK (though we have had visits from at least 40 countries). Over 200 web pages have links to NDTM and that number is rising.

**Discussion:**

The information obtained from this has proved very useful in developing the Web site. For example, the link that is most frequently followed to NDTM is from the NHS Executive IM&T Web site, indicating that our Web site has a large proportion of its readership within the UK health community. We have also been able to analyse the keywords that people use to find us using the Internet search engines, and revised the content of our pages to make our site easier to find. In the future, we may also use the information to target content at particular types of reader or to help in assessing where on the Internet to set up mirrors of the Web site.

